# Editorial: 3D printing and virtual surgery in veterinary dentistry and oromaxillofacial surgery

**DOI:** 10.3389/fvets.2026.1775641

**Published:** 2026-02-04

**Authors:** Lisa Alexandra Mestrinho, Ana C. Castejón-González

**Affiliations:** 1 Centre for Interdisciplinary Research in Animal Health (CIISA), Faculty of Veterinary Medicine, University of Lisbon, Lisbon, Portugal; 2 Department of Clinical Sciences and Advanced Medicine, School of Veterinary Medicine, University of Pennsylvania, Philadelphia, PA, United States

**Keywords:** 3D printing, computer-aided design, oromaxillofacial surgery, veterinary dentistry, virtual surgery

Tridimensional (3D) virtual simulation and additive manufacturing applications have brought transformative changes to veterinary dentistry and oromaxillofacial surgery. By creating 3D models, veterinarians can physically explore both normal and pathologic anatomy, practice surgical techniques, better anticipate challenges of an intervention, improve precision, and reduce both surgical time and surgical risk.

From the first published clinical case series (1, 2) to the development of structured methodological guidelines (3, 4), 3D-printing and virtual planning have shown consistent growth. These surgical planning modalities are now relatively well-established and are likely to become increasingly available to veterinarians and their patients (5).

This Research Topic includes seven peer-reviewed manuscripts exploring the subdisciplines of veterinary dentistry—surgery, endodontics, and prosthodontics—whereby 3D imaging data were used in virtual planning to analyze anatomy, evaluate and compare accuracy in prosthetic design, simulate access paths and fracture patterns; some ultimately resulting in 3D-printed guides, models or prosthodontic components.

Endodontic access was investigated in two studies focusing on more complex teeth of dogs, namely the maxillary fourth premolar tooth and mandibular first molar tooth. Endodontic access is a high-precision procedure that is strongly dependent on operator experience. Detailed knowledge of endodontic anatomy and optimal access angulation is therefore critical to procedural success; however, such information has been limited in the existing veterinary literature. Peng et al. proposed endodontic guides to improve procedural predictability in endodontic access of mandibular teeth. From the computed tomography (CT) scan of a dog, a technique was created to obtain a virtual model of the tooth, later printed along with a surgical drilling guide. Such drilling guides were tested *in vitro* using 3D-printed dental models and were compared with classic root canal access. The authors conclude that there were significant variations between both techniques and that surgical drilling guides can contribute to a reduction of endodontic access size and instrumentation complications including ledges and perforation of the chamber floor. Morin and D'Astous investigated the endodontic anatomy of the maxillary fourth premolar using micro-CT analysis and virtual planning, identifying clinically relevant variations. In particular, the mesiopalatal canal exhibited a marked curvature, with an average angulation of approximately 150 degrees in its coronal third. These findings provide new insight into the challenges of endodontic access and inform the ongoing discussion regarding the effectiveness of different access techniques. Despite their exploratory nature, such studies can contribute meaningfully to the optimization of endodontic access design and instrumentation strategies.

Virtual surgery planning was also a key focus in this Research Topic, applied to study mandibular fracture patterns in cats. Tu et al. and Castejón-Gonzalez et al. explored feline mandibular fractures from complementary perspectives, providing an overview of their anatomical complexity and highlighting the influence of regional mandibular anatomy. Their findings support the utility of virtual planning and 3D-printing in guiding fixation strategies and implant design. Castejón-Gonzalez et al. employed 3D printed models and identified an association between fracture patterns and fracture etiology; notably, at least half of the fractures were severely displaced, indicating the frequent need for more invasive stabilization techniques. Tu et al. used virtual fracture mapping in a separate case series, suggesting a potential role of morphological and geographic features of the mandibular angle in determining fracture patterns.

In the field of prosthodontics, the accuracy of digital impressions was compared with that of stone casts commonly used in clinical practice. The study by Metje et al. demonstrated that intraoral scanner systems can provide reliable and accurate results and may represent a more streamlined alternative in veterinary dentistry. Their findings indicated comparable accuracy between digital impressions and conventional stone models, offering workflow advantages and a reduction of operator-dependent variability in dental impressions. In a second study within this Research Topic, the same authors compared the accuracy of surface reconstructions derived from cone beam computed tomography (CBCT) datasets with that of conventional stone models for the same purpose. Given the widespread availability of CBCT in referral dental clinics, this approach represents a promising alternative for practitioners who do not have access to intraoral scanners. However, higher-resolution CBCT reconstruction algorithms are required to achieve comparable accuracy to stone models, and concerns related to radiation exposure and overall workflow efficiency remain to be addressed in future studies.

Finally, Nedelea et al. presented a multimodal investigation of a canine tooth from a jaguar exhibiting pink discoloration. In this post-mortem case study, computer-aided design and three-dimensional printing were used to create a prosthodontic replacement, allowing the specimen to be preserved and displayed in a zoological collection. The described procedure was particularly relevant to this Research Topic due to the unique size and anatomical characteristics of the tooth, demonstrating the adaptability of digital planning and additive manufacturing techniques to non-standard clinical and anatomical contexts.
